# Overdose reversal through peer intervention: Findings from the naloxone prospective outcome research study (NalPORS) on the effectiveness of overdose response by people who use opioids in the United Kingdom and Sweden

**DOI:** 10.1111/add.70526

**Published:** 2026-07-12

**Authors:** Nicola Metrebian, Teodora Dascal, Clare Mackie, Rose Tinch‐Taylor, Ben Carter, Joanne Neale, Mariana Gonzalez Utrilla, Ed Day, Joar Guterstam, Kirsten Horsburgh, Martin Kåberg, Mike Kelleher, Soraya Mayet, Josie Smith, Desiree Eide, Elin Holmén, Rebecca McDonald, Stephen Parkin, Henrik Thiesen, John Strang, NalPORS Research Team, Dean Acreman, Shabana Akhtar, Louise Aitken, Martin Anderson, James Cassidy, Sarah Cosgrove, Jo Grayer, Michelle Hughes, Louise Horn, Anika Miah, John Pratt

**Affiliations:** ^1^ National Addiction Centre, Institute of Psychiatry, Psychology and Neuroscience King's College London London UK; ^2^ Department of Biostatistics and Health Informatics, Institute of Psychiatry, Psychology and Neuroscience King's College London London UK; ^3^ Birmingham and Solihull Mental Health NHS Foundation Trust Birmingham UK; ^4^ Stockholm Centre for Dependency Disorders, Stockholm, Sweden, Department of Global Public Health Karolinska Institutet Stockholm Sweden; ^5^ Scottish Drugs Forum Glasgow UK; ^6^ Department of Clinical Neuroscience Centre for Psychiatry Research, Karolinska Institutet and Stockholm Health Care Services, Stockholm County Council Stockholm Sweden; ^7^ South London and Maudsley NHS Foundation Trust London UK; ^8^ Humber Teaching NHS Foundation Trust Hull UK; ^9^ Public Health Wales Cardiff UK; ^10^ Norwegian Centre for Addiction Research University of Oslo Oslo Norway; ^11^ Sundheds Team Copenhagen Denmark

**Keywords:** death, emergency, mortality, naloxone, opioid, overdose, resuscitation, take‐home‐naloxone

## Abstract

**Background/aims:**

Take‐home naloxone (THN) programmes have been introduced in many European countries to provide non‐medically trained people who are likely to witness an opioid overdose, including people who use opioids, with a naloxone kit and training. This study used data obtained from people supplied with THN and systematically followed up for 6 months to determine the proportion witnessing an overdose, administering naloxone and successfully reversing opioid overdose in real‐world settings. The study aimed to compare reports of witnessed opioid overdose between participants in treatment and not in treatment at the time of recruitment.

**Design:**

A multi‐country, observational prospective cohort study. Participants supplied with THN were asked to report any witnessed overdose events and participate in a systematic follow‐up over a 6‐month period.

**Setting:**

Participants were recruited from THN programmes at 22 drug treatment and harm reduction services in England, Wales, Scotland and Sweden.

**Participants:**

A total of 1025 people who used opioids and who were supplied with THN were recruited between June 2021 and November 2023, of whom 594 (58%) were successfully followed at 6 months. The participants were predominantly men (75%) and white (80%), with a mean age of 45.5 years (standard deviation 10).

**Measurement:**

Main outcomes: whether or not THN was administered at a witnessed overdose and overdose outcome where THN administered. Accurate recognition of opioid overdose, availability of naloxone and appropriate responses (including administering naloxone) to an opioid overdose were captured. Outcomes were compared between participants in treatment and not in treatment.

**Findings:**

Of the 594 followed over 6 months, 160 (27%) witnessed at least one overdose. The likelihood of witnessing an overdose was higher among participants not in treatment than participants in treatment [56% vs. 23%; odds ratio = 4.4; 95% confidence interval (CI) = 2.69–7.21; *P* = 0.001]. THN was administered at most witnessed overdose events (83%; 95% CI = 74–85). Seven deaths were reported (4.4%; 95% CI = 0.02–8.8) by participants to be due to an overdose. For five deaths, naloxone was administered but was believed by participants to have been administered after death. Ninety‐five percent (152) of participants were able to recognise one or more critical signs of overdose and respond appropriately. When naloxone was available, it was administered in almost all (90%; 142) emergency situations.

**Conclusions:**

In the United Kingdom and Sweden, people who use opioids appear to be likely to witness and recognise an opioid overdose, be the only person carrying naloxone at the overdose and be both willing and able to safely and effectively administer naloxone in a timely response.

Clinical trial registration details (if applicable): ClinicalTrials.gov Identifier: NCT05072249. Date of Registration: 8.10.2021.

## INTRODUCTION

Opioid overdose deaths remain a major challenge for public health policy world‐wide [1, 2, 3, 4, 5, 6]. Naloxone is an emergency medication that, with timely administration, has proven to be safe and effective in rapidly reversing opioid overdose and preventing opioid overdose fatal outcome [7, 8, 9].

Since the late 1990s, take‐home naloxone (THN) programmes have been introduced. The programmes provide naloxone and training in how to recognise an overdose, to call an ambulance, to administer naloxone and perform basic life support to non‐medically trained individuals who are most likely to observe opioid overdoses (including people who use opioids themselves, their family and carers) [8, 9, 10, 11]. In 2014, the World Health Organisation (WHO) launched guidelines on the community management of opioid overdose, recommending that ‘people likely to witness an opioid overdose should have access to naloxone and be instructed in its administration’ [12]. Individuals using heroin and other opioids have reported that they support the use of THN and would be willing to administer naloxone in an overdose situation [10, 13, 14, 15, 16].

THN programmes in Europe now include different naloxone products and routes of administration. Injectable naloxone has been approved for administration by non‐medically trained persons present at an overdose emergency [4, 5, 6]. In recent years, several concentrated naloxone nasal spray formulations have additionally been approved, potentially increasing acceptability and wider provision [16, 17, 18, 19]. For some countries (including Sweden) broad implementation of THN only occurred once concentrated naloxone nasal spray formulations appeared [20].

Although the efficacy of naloxone is well‐established [7, 11, 16, 17, 18, 19], the science and technology around this medication has been transferred for use in a new setting (the community) and by a different population (non‐medically trained individuals). The process of technology transfer has included changes to naloxone formulation, dose, product packaging and training. The nature of THN has changed and it is important to understand uptake, administration and outcomes in community settings.

A systematic review applying the Bradford Hill criteria to international evidence of THN concluded that THN leads to reduced heroin overdose mortality rates in the community with a low rate of adverse events [21]. Another study, based on Bradford Hill criteria, also found an association between reduced deaths from overdose and the implementation of THN programmes [22]. However, the majority of studies included in the review [21] relied on unsystematic follow‐up to capture overdose events, using participants' reports when they returned for replacement kits. Moreover, only overdose fatality and adverse events were captured, and the review did not differentiate between injectable and nasal naloxone. More recently, individual studies evaluating THN distribution programmes in Norway and Denmark have demonstrated that, where nasal naloxone was administered, overdose mortality rates are very low [23, 24]. However, these studies relied on self‐report from participants returning for replacement naloxone, and the study conducted in Norway only included an adapted pre‐filled syringe with nasal atomiser [23]. In contrast, the Naloxone Prospective Outcome Research Study (NalPORS) recruited individuals at the point they were supplied with either injectable or nasal naloxone and followed them up prospectively over a 6‐month period obtaining data on one witnessed overdose event.

NalPORS aimed to assess the effectiveness of overdose response and overdose reversal by non‐medically trained persons who were supplied with THN in community settings, including comparisons between participants in‐treatment and those not‐in‐treatment at the time of recruitment (see Supporting information and protocol paper) [25].

### Study objectives

The study objectives are to determine the proportion of participants who witnessed an overdose, administered naloxone and successfully reversed an opioid overdose in real‐world settings; to assess the accuracy of identifying a probable opioid overdose and the appropriateness of the overdose response; and to determine the proportion of fatal outcomes occurring within 12 hours following a witnessed opioid overdose.

This paper presents data on a subgroup of the NalPORS cohort, people who themselves use opioids and were supplied with THN. This group are both at risk of overdose and are often the first to witness or respond to an overdose, making them critical to THN programmes. The key comparison was between participants in‐treatment and not‐in‐treatment, at the time of the recruitment. Those not in treatment were more likely to be currently using illicit opioids and more likely to be socialising with other people using opioids. We expected that they would be more likely to witness an overdose and better prepared to respond to one.

## METHODS

### Study design

We designed a multi‐country prospective cohort study of non‐medically trained individuals supplied with THN from drug and alcohol harm reduction or treatment services across England, Wales, Scotland and Sweden, between 2021 and 2024. All participants were followed up for 6 months. Any participant who reported witnessing an opioid overdose in this 6‐month period was offered a structured interview about this witnessed overdose event (our main interest).

Our study looked to capture reports from participants across several European countries, which, during the 6‐month study, had been present at and could provide accounts to us of witnessed opioid overdoses. We also wished this to be reasonably balanced across nasal and injectable forms of naloxone.

### Setting

To include participants supplied with naloxone, we approached treatment and harm reduction sites located within countries that were providing THN, had a history of opioid overdose fatalities and had the highest rates of drug‐related mortality in Europe [5, 6]. Countries were chosen to provide us with different available forms of naloxone within our cohort, and an approximately equal split between those providing nasal and injectable forms of naloxone (see Supporting information for more information).

### Participants

Eligible participants included non‐medically trained persons supplied with THN at study sites. These comprised four groups: (1) patients/service users in treatment for opioid use disorder (in‐treatment); (2) individuals using opioids but not currently in treatment (not‐in‐treatment) (groups 1 and 2 referred to as ‘people who use opioids’ are the focus of this paper); (3) family members or friends (or other close contact) of person who uses opioids; and (4) staff working with individuals with opioid use disorder (including volunteers, health care workers, social care workers and drug workers).

Eligibility criteria included: member of at least one of the groups above (1–4); 18 years or older, supplied with THN at the time of enrolment [or can demonstrate that they have a supply of THN (in‐date) and have received training at time of enrolment]; have access to reliable mobile telephone and can present it; and willing to participate in follow‐up. Exclusion criteria included: needing an interpreter; and not willing or able to participate in 6‐month follow‐up.

Sample size justification and the main objective of NalPORS were to estimate the opioid overdose reversal rate by laypeople with high confidence. We estimated that 600 participants would achieve this, assuming a reversal rate of 10%. Among the subgroup of people who use opioids and were supplied with THN, we were only able to enrol 1025 people leading to 160 observed overdoses (see Study protocol) [25].

### Recruitment and procedures

All persons supplied with THN who were in the four recruitment categories were screened for eligibility for the study by staff at the participating drug treatment and harm reduction service. Consenting participants were required to send a text message to the study's automated telephone system to be formally enrolled into the study and to prompt the telephone system to send monthly reminder texts to their mobile phone.

At enrolment, participants completed a structured questionnaire to capture socio‐demographic characteristics, previous drug use and previous experience of witnessed or experienced overdoses, naloxone provision and training. Participants were reimbursed with a £5 shop voucher. This was later increased to £10 to promote recruitment. In addition, staff members provided information on the type of naloxone kit and training provided to the participant at the time of enrolment into the study.

Once enrolled, participants were sent monthly text messages over the study period to prompt them to contact research staff if they had witnessed an opioid overdose event, to complete a structured interview about this event (in‐person or via telephone). Staff that had regular contact with participants (including replenishment of THN) were asked to prompt participants. By minimising the time between a participant reporting an overdose and interviewing them, we hoped to reduce any recall issues. Interviewed participants were reimbursed with a £15 shop voucher.

At 6 months, all participants were contacted by telephone, by the research team, to take part in a 6‐month follow‐up structured interview about THN carriage, training and any witnessed opioid overdose not previously reported. Participants were reimbursed with a £15 shop voucher. We later reduced this to £10 when we increased the reimbursement at enrolment to ensure the total amount participants received if they attended all interviews remained at £35.

### Outcomes

#### Main outcomes

The main outcomes included:

1. The proportion of participants supplied with THN, who witnessed an opioid overdose and administered naloxone with the intention of reversing an opioid overdose; and
2. The frequency of deaths in the 24 hours (or later when information was available) subsequent to the administration of naloxone.

#### Other outcomes

Other outcomes included:

1. Percentage of participants who witness an opioid overdose over 6‐month period;
2. Percentage of participants who state there was THN present and administered THN;
3. Percentage of participants who report fatal outcomes within 2 hours of the identification of opioid overdose or at the arrival of an ambulance/medical assistance where THN was not administered;
4. Percentage of participants who correctly diagnose opioid overdose crisis by identifying from a list of possible responses at least one key critical sign that this was an opioid overdose (critical signs include: loss of consciousness, unresponsiveness/lack of response to calling name or shaking or pain, shallow/absent/slow breathing, blue lips, hands or feet); and
5. Percentage of participants who appropriately respond to the overdose, in addition to administering naloxone, by identifying from a list of possible responses at least one appropriate response/intervention, including: tried to wake them physically, tried to wake them verbally, checked for response, checked breathing, ambulance called, put into recovery position, chest compressions, tilted head back, rescue breathing/mouth to mouth, checked pulse and cleared airways.

Data were captured through a structured questionnaire developed specifically for this study (see Supporting information).

### Analysis

Demographic and clinical characteristic data for study participants were reported for continuous variables (means and SD or medians and interquartile ranges) or frequencies and percentages for categorical variables. Descriptive data were reported as numeric counts and percentages with the denominator for reporting percentages appropriate to the specific question. χ^2^ tests of independence were conducted when examining between group differences for categorical variables and independent *t* tests for means. The main outcomes were administration rate of THN by people who use opioids witnessing an opioid overdose and the frequency of deaths in the 24 hours after administration of naloxone were estimated as proportions. All other outcomes were estimated as proportions. Where appropriate, 95% CIs are calculated for estimates of means and proportions. Missing data are reported. The data were collated within a REDCap database and analysed using Stata version 18 (StataCorp; College Station, Texas).

### Ethics approval

This study received United Kingdom (UK) National Health Services (NHS) Research Ethics Committee approval on 25 June 2020 from North‐East Newcastle and North Tyneside Research Ethics Committee (20/NE/0131) and Swedish Ethical Review Authority approval on 4 October 2021 (2021–04751).

## RESULTS

Between 8 June 2021 and 31 March 2024, 1028 individuals in the NalPORS subgroup, people who use opioids and supplied with THN, were enrolled in the study through 22 drug treatment and harm‐reduction services across four of the six intended countries: England, Wales, Scotland and Sweden. Three participants' data had to be excluded from the analyses because of missing consent forms (see Participant Flow diagram).

Despite the lower‐than‐anticipated recruitment, we successfully collected accounts of witnessed overdose events, with 160 participants reporting that they had experienced a witnessed opioid overdose event during the study period. Recruitment challenges included, at the country level: insecurity of funding for THN services, low numbers of reported opioid overdoses and absence of THN programmes; and at the site level: difficulties recruiting participants because of limited resources in part because of substantial service disruption during and following the coronavirus disease 2019 pandemic (see Supporting information).

### Characteristics of the participants who use opioids

Participants who use opioids were characterised into two groups, 902 (88%) patients/service users who were currently receiving treatment for opioid use disorder (‘in‐treatment’) and 123 (12%) individuals who use opioids (non‐prescribed) and were not currently receiving treatment (‘not‐in‐treatment’). Of these, the majority (59%) were recruited from England. Two‐thirds (67%) were recruited from drug treatment services and one‐third (33%) from harm reduction services. Most in‐treatment participants (75%) were recruited from drug treatment services. Almost all (94%) not‐in‐treatment participants were recruited from harm reduction services [e.g. needle and syringe programmes (NSP)]. A few were recruited from treatment services with an NSP. The majority (67%) of in‐treatment participants were recruited from England, and the majority (56%) of not‐in‐treatment participants were recruited from Sweden.

Most participants were male (74%), had a mean age of 44 years and 80% self‐reported their ethnicity as white. A greater proportion of in‐treatment participants were male (75% versus 64%) and slightly older (mean 45 versus 42 years) than not‐in‐treatment participants (see Supporting information).

At enrolment, 63% participants were supplied with (or already had) injectable naloxone (Prenoxad, a multi‐dose 2 mg injectable form of naloxone in a pre‐filled syringe), and 39% were supplied with (or already had) nasal naloxone. Those supplied with nasal naloxone included, 220 supplied with Nyxoid (a twin‐pack form of nasal naloxone 2 × 1.8 mg) and 176 supplied with Ventizolve (a twin‐pack form of nasal naloxone 2 × 1.26 mg) (see Table 1). Some participants were supplied with multiple kits and both injectable and nasal naloxone. Two‐thirds (67%) of in‐treatment participants were supplied with injectable naloxone and one‐third nasal naloxone (36%). In contrast, two‐thirds of not‐in‐treatment participants were supplied with nasal naloxone (62%) and one‐third injectable naloxone (37%).

**TABLE 1:** Characteristics of cohort of people who use opioids and followed‐up (*n* = 594) and participants who witnessed an overdose (witnessed overdose cohort) (*n* = 160).

	Total cohort of people who use opioids and followed‐up (*n* = 594)			Witnessed overdose cohort (*n* = 160)		
	Total people who have used opioids groups 1 and 2 *n* = 594, *n* (%)	Group 1 in treatment (patient/service user in treatment for opioid use disorder) *n* = 515, *n* (%)	Group 2 out of treatment (individual uses opioids and not currently in treatment) *n* = 79, *n* (%)	Total people who have used opioids groups 1 and 2 *n* = 160, *n* (%)	Group 1 in treatment (patient/service user in treatment for opioid use disorder) *n* = 116, *n* (%)	Group 2 out of treatment (individual uses opioids and not currently in treatment) *n* = 44, *n* (%)
Witnessed overdoses						
Rate of witnessed overdose	160/589 (27.2)	116/511 (22.7)	44/78 (56.4)			
Missing	5	4	1			
Number of witnessed overdoses				278 (range 1–20)	136 (range 1–20)	70 (range 1–8)
Countries						
England	338 (56.9)	335 (65)	3 (3.8)	51 (31.9)	49 (42.2)	2 (4.5)
Sweden	171 (28.8)	114 (22.1)	57 (72.2)	68 (42.5)	37 (31.9)	31 (70.5)
Scotland	46 (7.7)	38 (7.4)	8 (10)	24 (15)	17 (14.7)	7 (15.9)
Wales	39 (6.6)	28 (5.4)	11 (14)	17 (10.6)	13 (11.2)	4 (9.1)
Type of recruitment site						
Harm reduction	208 (35)	132 (25.6)	76 (96.2)	98 (61.3)	56 (48.3)	42 (95.5)
Treatment service	386 (65)	383 (74.4)	3 (3.8)	62 (38.8)	60 (51.7)	2 (4.5)
Participant characteristics						
Age at enrolment	45.5; SD 10 (19.5–71)	46; SD 9.8 (21.4–71)	42.3; SD 10.9 (19.5–69)	42.4; SD 10.3 (19.5–69)	42.6; SD 10.2 (21.8–64.2)	41.8; SD 10.7 (19.5–69)
Gender						
Male	443 (74.9)	391 (76.5)	52 (65.8)	111 (69.8)	82 (71.3)	29 (66.9)
Female	148 (25)	121 (23.5)	27 (34.2)	48 (30.2)	33 (28.7)	15 (34)
Missing	3	3	0	1	1	0
Ethnicity						
White	472 (79.6)	406 (79)	66 (83.5)	129 (80.6)	92 (79.3)	37 (84.1)
Black/African/Caribbean/Black British	41 (6.9)	39 (7.6)	2 (2.5)	9 (5.6)	8 (6.9)	1 (2.3)
Mixed/multiple ethnic group	36(6.1)	29 (5.6)	7 (8.9)	10 (6.3)	6 (5.2)	4 (9.1)
Other ethnic groups	26 (4.4)	26 (5.1)	0	4 (2.5)	4 (3.4)	0 (0)
Asian/Asian British	14 (2.4)	11 (2.1)	3 (3.8)	6 (3.8)	4 (3.4)	2 (4.5)
Prefer not to say	4 (<1)	3 (<1)	1 (1.3)	2 (1.3)	2 (1.7)	0 (0)
Missing	1	1	0			
Type of naloxone kit supplied at enrolment						
Injectable naloxone: pre‐filled syringe (Prenoxad)	346 (56.8)	326 (61.4)	20 (25.3)	77 (48.1)	65 (56)	12 (27.3)
Nasal naloxone (Nyxoid and Ventizolve)	254 (41.6)	195 (36.7)	59 (74.7)	84 (52.5)	52 (44.8)	32 (72.7)
Other type of naloxone	10 (1.6)	10 (1.9)	0	4 (2.5)	4 (3.4)	0

Of the 1025 people who use opioids, 594 were successfully followed up at 6‐months or completed a witnessed overdose interview before their 6‐month research interview (58%; 95% CI = 55–61). Follow‐up rates were lower than non‐follow‐up rates in England, Scotland and Wales, but higher in Sweden. The characteristics of participants followed‐up (*n* = 594) and not followed up (*n* = 431) were similar (see Supporting information).

### Characteristics of people who use opioids and witnessed an overdose within 6 months

Of those followed‐up 160 (27%) reported that they had witnessed at least one overdose (and a total of 278 overdoses) in the 6‐month study period. Compared to in‐treatment participants, not‐in‐treatment participants were four times more likely to report witnessing an overdose (23% versus 56%) (OR = 4.4; 95% CI = 2.69–7.21; *P* < 0.001). Similarly, participants recruited from harm reduction sites were more likely to report witnessing an overdose compared to those recruited from treatment sites (61% versus 39%) (χ^2^ = 29.92; d.f. = 1, *P* < 0.001). The number of participants who had witnessed at least one overdose varied by country.

Among the 160 people who had witnessed an overdose within the 6 months study period, 70% were male, 81% self‐reported white ethnicity and their mean age was 42 years (range = 19.5–69). Compared to the wider cohort of people who use opioids, were supplied with THN and followed‐up (*n* = 594), a larger proportion of those witnessing an overdose were recruited from harm reduction services (35% versus 61%) and were female (25% versus 30%) (Table 1).

### Characteristics of the opioid overdose event

People who use opioids and witnessed an overdose event (*n* = 160), reported witnessing an overdose at a range of locations, including a public setting (e.g. shopping centre) (38%), someone else's home (33%) and in the participants own home (15%) (Table 2). The relationship between the participant who witnessed the overdose and the person experiencing the overdose was most likely to be one of friend (46%) or acquaintance (39%) with 11% being partner or spouse. Participants in‐treatment and not‐in‐treatment groups reported similar locations and relationships.

**TABLE 2:** Characteristics of witnessed overdose event (*n* = 160).

Witnessed overdose	Total people who have used opioids groups 1 and 2 *n* = 160, *n* (%)	Group 1 in‐treatment (patient/service user in treatment for opioid use disorder) *n* = 116, *n* (%)	Group 2 out of treatment (individual uses opioids and not currently in treatment) *n* = 44, *n* (%)
Relationship to person experiencing OD			
Friend	66 (45.5)	49 (46.7)	17 (42.5)
Acquaintance	57 (39.3)	39 (37.1)	18 (45)
Partner/spouse	16 (11)	11 (10.5)	5 (12.5)
Other relationship	6 (3.8)	6 (5.2)	0
Missing	15	11	4
Location of OD			
Public setting (e.g. shopping centre)	60 (37.7)	41 (35.7)	19 (43.2)
Someone else's home	52 (32.7)	40 (34.8)	12 (27.3)
At my home	23 (14.5)	15 (13)	8 (18.2)
Hostel	9 (5.7)	8 (7)	1 (2.3)
Other	15 (9.4)	11 (9.5)	0
Missing	1	1	0
Drugs used			
Heroin	142 (94.7)	104 (94.5)	38 (95)
Street benzodiazepines (e.g. Xanax, Valium)	57 (38)	36 (32.7)	21 (52.5)
Crack cocaine	35 (23.3)	34 (30.9)	1 (2.5)
Alcohol	35 (23.3)	28 (25.5)	7 (17.5)
Tobacco	19 (12.7)	11 (10)	8 (20)
Other non‐opioids (amphetamine, cannabis, spice)	20 (12.5)	15 (12.9)	5 (11.4)
Prescribed opioids (methadone, buprenorphine, other)	18 (11.3)	14 (12.1)	4 (9.1)
Other illicit opioids (fentanyl, oxycodone, non‐prescribed methadone or buprenorphine, other)	10 (3.1)	9 (7.75)	1 (2.27)
Other	6 (4)	5 (5.4)	
Missing	10 (6.7)	6 (5.5)	4 (10)

Abbreviation: OD, overdose.

Participants were asked whether they knew which drugs the person experiencing the overdose had used. All (100%) reported that they believed opioids had been used with heroin reported (95%) in the majority of cases. Other drugs reported to have been used in addition to opioids included: street benzodiazepines (38%), crack cocaine (23%) and alcohol (23%) (Table 2).

### Main outcomes

#### Proportion of people who use opioids, who witnessed an opioid overdose and administered naloxone

Among the 160 participants who witnessed an overdose in the study period, 83% (95%CI = 74–85) reported that naloxone was administered at the witnessed overdose event. Of the 128 instances when naloxone had been administered, 77% of participants reported administering the naloxone themselves (Table 3).Compared to participants in‐treatment, a larger proportion of participants not‐in‐treatment reported that naloxone was administered at the overdose event (76% versus 91%) and that it was administered by themselves (59% versus 86%).

**TABLE 3:** Percentage of participants witnessing an overdose who reported naloxone was available and administered (*n* = 160).

	Total people who have used opioids groups 1 and 2 *n* = 160, *n* (%)	Group 1 in‐treatment (patient/service user in treatment for opioid use) *n* = 116, *n* (%)	Group 2 out of treatment (individual uses opioids and not currently in treatment) *n* = 44, *n* (%)
Was naloxone available?			
Yes	142 (90.4)	100 (87.7)	42 (97.7)
No	15 (9.6)	14 (12.3)	1 (2.3)
Missing	3	2	1
If yes, whose naloxone was available?(*n* = 142)			
Mine	97 (61.8)	67 (58.8)	30 (69.8)
Someone else's	27 (17.2)	20 (17.5)	7 (16.3)
Mine and someone else's	18 (11.4)	13 (11.4)	5 (11.6)
Did you or someone else administer naloxone?			
Yes	128 (82.6)	88 (79.3)	40 (90.9)
No	27 (17.4)	23 (20.7)	4 (9.1)
Missing	5	5	0
If yes, who administered naloxone? (*n* = 128)	e		
Yes, me	99 (63.9)	69 (62.2)	30 (68.2)
Yes, someone else	29 (18.7)	19 (17.1)	10 (22.7)
If naloxone was administered, which type of naloxone was used? (*n* = 128)			
Nasal naloxone	67 (52)	39 (45.3)	28 (73.70)
Injectable naloxone	61 (48)	51 (59.3)	10 (26.3)
Other	1 (<1)	1 (1.2)	0
Missing	4	2	2

At the witnessed overdose event, where naloxone was administered (*n* = 128), 54% participants reported that nasal naloxone was administered and 49% that injectable naloxone was administered. Some participants reported that both nasal and injectable naloxone was administered (Table 3). Not‐in‐treatment participants were more likely to report nasal naloxone was administered, than in‐treatment participants (74% versus 45%; χ^2^ = 9.97, d.f. = 1; *P* = 0.002). These proportions reflect the type of naloxone supplied at enrolment.

Of those participants witnessing an overdose (*n* = 160), 27 reported that naloxone was not administered at the overdose event. In 15 reported witnessed overdose events, naloxone was not available. When naloxone was available at the overdose event, but not administered (*n* = 12), reasons for not administering it (from a checklist of possible responses) included, ‘I judged it was not necessary at that point in time’(*n* = 3), ‘person recovered’(*n* = 2), ‘didn't have it on me’ (not carrying own THN) (someone else's naloxone kit was available at the overdose emergency) (*n* = 2), ‘no longer possessed a THN kit (lost or previously used)’ (*n* = 1), ‘panicked’ (*n* = 1) ‘someone else intervened’ (*n* = 1) (Table 3).

#### Frequency of fatal outcomes

Of the 160 participants who witnessed an overdose, 151 reported on the outcome of the overdose (9 cases unknown). Of these, 143 (94.7%) participants reported that the person experiencing the overdose was still alive at the time of being interviewed. In total, seven (7/151) fatalities were reported by participants who witnessed an opioid overdose (4.6%; 95% CI = 0.02–8.8). All were because of an opioid overdose (Table 4).

**TABLE 4:** List of fatal outcomes among individuals experiencing an overdose and receiving THN.

Country	Who provided naloxone?	How soon after the overdose did the person die?	Cause of death
Wales	Yes, me	Within 2 hours	Opioid overdose^a^
Scotland	Yes, me	Unknown/I do not know	Opioid overdose^a^
Sweden	Yes, me	Within 2 hours	Opioid overdose^a^
Sweden	Yes, me	Within 2 hours	Opioid overdose^a^
England	Yes, me	Unknown	Opioid overdose^a^ ^,^ ^b^
England	Yes, me	Unknown	Opioid overdose^a^ ^,^ ^b^

Abbreviation: THN, take‐home naloxone.

^a^ Death had likely occurred before first naloxone administration.

^b^ Two participants witnessed the same opioid overdose and hence both reported the same death.

Two (2/27) fatalities (7.4%; 95% CI = 0.8–20.8) occurred where naloxone had not been administered. Six participants who witnessed an overdose where naloxone was administered (*n* = 128), reported five deaths (3.9%; 95% CI = 1.28–8.9). Two participants had witnessed the same opioid overdose and reported the same death. However, all five fatalities were reported by the participant to be because of a drug overdose, and none were considered by the participant to be because of the naloxone administered. In all five cases, the study participant reported that either they or the emergency services believed the death occurred before naloxone administration. Participants reported that three deaths occurred within 2 hours of the overdose. The time of death for two was unknown.

### Other outcomes

#### Able to correctly identify an opioid overdose emergency

Participants who reported witnessing an overdose were asked to identify from a list of possible responses what were the signs that made them or someone else recognize that this was an opioid overdose including: (1) key critical signs; (2) other signs; and (3) miscellaneous signs/not signs of opioid overdose, but might be signs that something is wrong. The majority (95%) of participants were able to recognize one or more critical signs of an opioid overdose crisis. Similar large majorities of in‐treatment (95%) and not‐in‐treatment (95%) participants recognised at least one critical sign (Table 5).

**TABLE 5:** Percentage of participants who witness an overdose who report signs that made them or someone else recognise the opioid overdose (witnessed overdose cohort *n* = 160).

	Total people who have witnessed an overdose groups 1 and 2 *n* = 160, *n* (%)	Group 1 in‐treatment (patient/service user in treatment for opioid use disorder) *n* = 116, *n* (%)	Group 2 not‐in‐ treatment (individual uses opioids and not currently in treatment) *n* = 44, *n* (%)
Critical signs			
Recognised one or more critical signs	152 (95)	110 (94.8)	42 (95.5)
Unresponsive/lack of response to calling their name or to shaking or pain	122 (76.3)	83 (71.6)	39 (88.6)
Loss of consciousness	105 (65.6)	84 (72.4)	21 (47.7)
Shallow/absent/slow breathing	85 (53.1)	58 (50)	27 (61.4)
Blue lips, hands or feet	84 (52.5)	61 (52.6)	23 (52.3)
None recorded	8 (5.1)	6 (5.2)	2 (4.5)
Other signs			
Recognised one or more other signs	78 (48.8)	60 (51.7)	18 (40.9)
Position of body	46 (28.8)	36 (31)	10 (22.7)
Deep snoring/grunting	32 (20)	24 (20.7)	8 (18.2)
Collapse	18 (11.25)	17 (14.7)	1 (2.3)
Needle in arm	10 (6.3)	9 (7.8)	1 (2.3)
Small pupils	6 (3.8)	4 (3.4)	2 (4.5)
Presence of vomit	1 (<1)	1 (<1)	0
None recorded	1 (<1)	1(<1)	0
Miscellaneous signs			
Presence of drooling	7 (4.4)	7 (6)	0
Fitting	4 (2.5)	4 (3.4)	0
Person reported not feeling right	2 (1.3)	2 (1.7)	0
Agitated behaviour	1 (<1)	0	1 (2.3)
Bloodshot eyes	1(<1)	1 (<1)	0
Rapid heartbeat	1(<1)	1 (<1)	0
Other	9 (5.6)	9 (7.8)	0

#### Availability of naloxone at the witnessed opioid overdose

Of the 160 participants who witnessed an overdose, 90% reported that naloxone was available at the witnessed overdose event. Where naloxone was available, 62% of participants reported that only their own naloxone kit was available. There was no difference in availability of kit or whose kit was available between in‐treatment and not‐in‐treatment participants (see Table 2).

#### Responding to an opioid overdose

Participants who witnessed an overdose were asked whether they or someone else did anything, in addition to administering naloxone, to respond to the overdose emergency. They were asked to identify appropriate actions from a list of fixed responses. To capture possible inappropriate responses, a list of possible inappropriate responses to an opioid overdose was compiled, but was not read out to the participant (Table 6). A large majority (97.5%) reported at least one ‘appropriate response’ was undertaken. The most frequently reported ‘appropriate responses’ included: ‘tried to wake them physically’ (63%), ‘tried to wake them verbally’ (59%) and ‘checked for a response’ (55%). Similar responses were reported by in‐treatment and not‐in‐treatment participants. A few participants (*n* = 9) reported an ‘inappropriate response’ of ‘walking person around’, while four participants reported providing a harmful response by ‘injecting them with a stimulant drug’. Only two participants reported that they or someone else did nothing to respond to the overdose.

**TABLE 6:** Percentage of participants witnessing an overdose who reported whether they or someone else did anything to respond to the overdose (*n* = 160).

Did you (or someone else) do anything to respond to the overdose?	Total people who have used opioids and witnessed an overdose groups 1 and 2 *n* = 160, *n* (%)	Group 1 in‐treatment (patient/service user in treatment for opioid use) *n* = 116, *n* (%)	Group 2 out of treatment (individual uses opioids and not currently in treatment) *n* = 44, *n* (%)
Appropriate response			
Responded with one or more appropriate responses	153 (95.6)	109 (94)	44 (100)
Tried to wake them physically	100 (62.5)	70 (60.3)	30 (68.2)
Tried to wake them verbally	95 (59.4)	67 (57.8)	28 (63.6)
Checked for response	88 (55)	64 (55.2)	24 (54.5)
Checked breathing	77 (48.1)	58 (50)	19 (43.2)
Ambulance called	68 (42.5)	52 (44.8)	16 (36.4)
Put into recovery position	63 (39.4)	47 (40.5)	16 (36.4)
Chest compressions	36 (22.5)	23 (19.8)	13 (29.5)
Tilted head back	35 (21.9)	25 (21.6)	10 (22.7)
Rescue breathing/mouth to mouth	28 (17.5)	17 (14.7)	11 (25)
Checked pulse	19 (11.9)	14 (12.1)	5 (11.4)
Cleared airways	13 (8.1)	12 (10.3)	1 (2.3)
Ambulance gave naloxone	16 (10)	13 (11.2)	3 (6.8)
I asked if they wanted me to use naloxone	7 (4.4)	5 (4.3)	2 (4.5)
Inappropriate response			
Walking person around	9 (5.6)	6 (5.2)	3 (6.8)
Injecting a stimulant drug	4 (2.5)	3 (2.6)	1 (2.3)
No response			
Nothing	2 (1.3)	1 (<1)	1 (2.3)
Other	6 (3.8)	6 (5.2)	0

## DISCUSSION

In this multi‐national, prospective cohort study with active follow‐up of people who use opioids and were supplied with THN, we found that naloxone was almost always available at a witnessed opioid overdose event, and when available, was almost always successfully administered to reverse an opioid overdose. There were only a few cases where naloxone was available and not administered.

We found much higher rates of witnessed overdose (27%) in the 6‐month follow‐up than we had anticipated from the previous literature (for our provisional power calculations, we had assumed a frequency of 10% of the sample being present at an overdose [25]). In addition, the 160 participants who had witnessed at least one opioid overdose had actually witnessed a total of 278 opioid overdoses within a 6‐month period. Using a systematic follow‐up, our study provides better estimates of witnessed overdose, which will have implications for THN provision and estimating sample size calculations for future research.

Naloxone was administered in most witnessed overdoses (83%) and where naloxone was administered, it was most often administered by one of our cohorts of people who use opioids (77%) rather than another bystander. Studies in Europe have reported lower rates of administration, including research in Norway examining people who use opioids and were supplied with THN and returning for refills (53%) [23, 26] and a European survey of people considered to be potential overdose witnesses (20%) [27]. Similar low rates among similar populations have been reported in the United States (23.2%; 16.9%) [28, 29]. Using a systematic follow‐up design may have provided better estimates of the administration of naloxone.

Fatal outcome was reported in one in every 23 witnessed overdose cases. Although this is similar to the one in every 20 to 30 overdose events previously reported in 2003 [30], it is much higher than the one in 123 overdose cases reported in a 2016 systematic review [21]. Naloxone was successfully administered in 96% of witnessed overdose events. In the five cases where the person experiencing the overdose died and naloxone was administered, participants witnessing the overdose believed that naloxone had been administered after death. A similar overdose survival rate of 83% to 100% has been reported in a previous systematic review of THN programmes [31].

A key part of administering naloxone is being able to recognize an opioid overdose. In the community, those witnessing an overdose may not know the person experiencing the overdose and may not know what drugs they have taken or in what quantities. We considered the presence of even one key critical diagnostic sign of an opioid overdose is sufficient for a conclusion of effective ‘diagnosis’ and enough to prompt appropriate action, including naloxone administration, by emergency services. We found most participants (95%) identified at least one critical sign indicating good knowledge of the signs of an opioid overdose emphasised in overdose response training.

There was a similar almost equal split between injectable and nasal naloxone supplied to those witnessing an overdose and administered at the overdose, reflecting different practices of different services, not the participants themselves. Those not‐in‐treatment participants were more likely to have been supplied with nasal naloxone and, therefore, to report use of nasal naloxone at a witnessed overdose. The type of naloxone offered by the treatment or harm reduction service is influenced in some settings by the law (e.g. only nasal naloxone is available on the market in Sweden) or because of funding considerations because the cost of nasal naloxone is higher in the United Kingdom.

Effective opioid overdose response requires the administration of naloxone and basic first aid. Most participants reported appropriate responses in line with THN training, with no differences between in‐treatment and not‐in‐treatment participants. These findings echo those of previous research and WHO guidelines demonstrating that people who use opioids can respond appropriately and effectively to an opioid overdose [12, 32, 33, 34, 35, 36]. However, fewer than half (43%) reported that they or someone else called an ambulance, a concerning low rate, given its emphasis in overdose training. In a linked qualitative study, interviews with people who used opioids and witnessed an overdose revealed that some people did not call an ambulance because of concerns that this might trigger the police to arrive at the overdose scene [37]. Similar low rates of calling an ambulance and concerns around a police response have been reported over the last two decades [31, 38, 39]. It is disappointing to find the same concerns still reported two decades later.

There was much variation in witnessed overdose rates between countries, with Scotland, Wales and Sweden all having high rates compared with England. One possible reason for such variation is likely to be because of country variations in opioid use (including types of opioids supplied and consumed), opioid overdose rates, overdose prevention initiatives and type of recruitment sites. As a marker for opioid overdose rates, rates of fatal opioid overdose are high in Scotland, Wales and Sweden [40, 41, 42, 43, 44]. Country difference may also be because of the mix of harm reduction and treatment sites across different countries. Higher overdose rates were reported among not‐in‐treatment compared to in‐treatment participants and those recruited from harm reduction services compared to drug treatment services. There were no participating harm reduction sites in England.

### Implications for practice

Our findings highlight the need for harm reduction services to deliver THN programmes to opioid users not‐in‐treatment who are witnessing a high number of opioid overdoses and are themselves at a high risk of experiencing an overdose. At the witnessed overdose event where naloxone was available, the kit usually belonged to these participants. This further highlights the importance of ensuring that people who are supplied with THN carry their naloxone kit on them, particularly as one‐third of witnessed overdoses took place in a public setting and one‐third in someone else's home. Those provided with THN should receive training in basic first aid, including calling an ambulance with staff addressing common concerns/barriers to calling emergency services.

### Strengths and limitations

A prospective cohort design is a strong methodology for this research and has enabled us to collect data shortly after a witnessed overdose event or in real time, which improves the accuracy of information regarding naloxone administration and overdose outcomes. This design has also allowed us to establish the temporal sequence between the provision of naloxone kits and training and subsequent overdose events. By following participants over time, we have been able to assess whether naloxone was available and successfully administered during an overdose and what the resulting outcomes were. As a result, this prospective cohort study provides more reliable evidence on the effectiveness and real‐world use of naloxone. Moreover, the nature of THN has changed, and this study provides a better understanding of uptake, administration and outcomes in a community setting.

Evaluating THN programmes is challenging, particularly conducting a 6‐month prospective cohort study of individuals at high risk of witnessing an overdose because of participant follow‐up difficulties. We achieved a 58% follow‐up rate, which we consider good given the challenges of maintaining contact over 6 months with a population that may not be in treatment, may have unstable or transient housing and frequently change mobile phones. In addition, treatment and harm reduction services experience high attrition rates among this population [43, 45, 46]. Nevertheless, we successfully followed‐up individuals not‐in‐contact with treatment services and found they encountered substantially more overdose reversal opportunities. Future studies should ensure inclusion of this wider population in the design and conduct of such studies.

Data are self‐reported and subject to recall bias, which we mitigated by conducting interviews as close as possible to the overdose event. Participants may not have known the person experiencing the overdose and may have lacked some information. Additional potential bias arises from reliance on self‐report overdose outcomes. It is possible that only individuals with successful overdose response experiences, in which the person survived, reported witnessing an overdose. Those with negative experiences might have failed to contact researchers.

## CONCLUSIONS

People who use opioids are likely to witness and recognise an opioid overdose, be the only person carrying naloxone at the overdose and be both willing and able to safely and effectively administer naloxone in a timely response. Our study is unique in its large‐scale prospective design and has provided better estimates of witnessed overdoses and the administration of naloxone.

These findings strongly support further public health measures aimed at increasing access to THN among people who themselves use opioids who are likely to witness an overdose in community settings.

## AUTHOR CONTRIBUTIONS

**Nicola Metrebian:** Writing—original draft; writing—review and editing; project administration; supervision; methodology; funding acquisition. **Teodora Dascal:** Investigation; writing—review and editing; project administration; data curation; methodology; software; visualization. **Clare Mackie:** Investigation; writing—review and editing; methodology; validation; formal analysis; project administration. **Rose Tinch‐Taylor:** Formal analysis; writing—review and editing. **Ben Carter:** Formal analysis; funding acquisition; writing—review and editing; methodology. **Joanne Neale:** Funding acquisition; writing—review and editing; supervision; methodology. **Mariana Gonzalez Utrilla:** Writing—review and editing; formal analysis; methodology. **Ed Day:** Writing—review and editing; methodology; supervision. **Joar Guterstam:** Writing—review and editing; methodology; supervision. **Kirsten Horsburgh:** Writing—review and editing; supervision; methodology. **Martin Kåberg:** Writing—review and editing; supervision; methodology. **Mike Kelleher:** Methodology; supervision; writing—review and editing. **Soraya Mayet:** Writing—review and editing; supervision. **Josie Smith:** Supervision; methodology; writing—review and editing. **Desiree Eide:** Investigation; methodology; writing—review and editing; project administration. **Elin Holmén:** Investigation; writing—review and editing; supervision; methodology. **Rebecca McDonald:** Investigation; writing—review and editing; project administration; methodology. **Stephen Parkin:** Investigation; writing—review and editing; methodology. **Henrik Thiesen:** Methodology; writing—review and editing. **John Strang:** Conceptualization; funding acquisition; writing—review and editing; methodology; supervision.

## DECLARATION OF INTERESTS

N.M. has secured, through her university, research funding from a pharmaceutical company, Mundipharma Research (for research on naloxone). She has also worked, through her university, with technology companies on research funded by the National Institute for Health and Care Research (NIHR). In the last 3 years, J.N. has secured, through her university, research funding from Mundipharma Research (for research on naloxone) and Camurus AB (for research on long‐acting injectable buprenorphine). She has also received honoraria from Indivior and Camurus AB for presentations and dissemination activities relating to drug treatment, from Figure 8 Consultancy for research advice on a study of residential rehabilitation, from the journals *Addiction* and the *International Journal of Drug Policy* for editorial work, and from the Canadian Centre for Recovery Excellence (CoRE) for participating in an Expert Reference Group for a national survey of recovery from alcohol and other drug use. M.K. has received honoraria for lectures from Camurus, DnE Pharma, Gilead, Mundipharma and Nordic Drugs and has received research grants from Gilead. M.K. has in the last 10 years been Chief or principle Investigator in Studies of pharmacotherapy funded by Camurus, Indivior, Beckley Tech, Mundipharma and the NIHR. J.S. is a clinician and academic who has worked with local, national and international government and non‐government agencies to develop and test new approaches to tackling addiction and related problems. Through his employer (King's College London), he has received research and project grant support from a range of government and charitable research agencies and charitable organisations. Through the university, he has also worked with pharmaceutical and technology companies from some of whom the university has received project grant support and/or honoraria and/or consultancy payments, as described at https://www.kcl.ac.uk/people/john-strang (including, past 3 years, Mundipharma, Camurus, Pneumowave, Accord and Navamedic) and also medication or device supply (Pneumowave, CMI and Catalent) to develop or study potentially improved formulations and devices. His employer (King's College London) previously registered intellectual property on an innovative buccal naloxone that J.S. is testing, and he has previously been named in a patent registration by a Pharma company as inventor of concentrated naloxone nasal spray. He is also named as a Patron of Addiction Family Support (formerly DrugFAM), a UK‐based registered charity. J.S. is supported by the NIHR Biomedical Research Centre (BRC) for Mental Health at South London and Maudsley NHS Foundation Trust and King's College London. T.D., E.D., C.M., R.T.T., B.C., M.G., J.G., S.M.D.A., S.A., L.A., M.A., J.C., S.C., D.., J.G., K.H., E.H., M.H., L.H., A.M., R.M., S.P., J.P., J.S. and H.T. have no declarations of competing interest.

## NalPORS RESEARCH TEAM

Dean Acreman, Shabana Akhtar, Louise Aitken, Martin Anderson, James Cassidy, Sarah Cosgrove, Jo Grayer, Michelle Hughes, Louise Horn, Anika Miah and John Pratt.

## Supporting information


**Data S1.** Supporting Information.

**Data S2.** Supporting Information.

## Data Availability

The data that support the findings of this study are available on request from the corresponding author. The data are not publicly available due to privacy or ethical restrictions.
