# Supplementary material for: Overdose reversal through peer intervention: Findings from the naloxone prospective outcome research study (NalPORS) on the effectiveness of overdose response by people who use opioids in the United Kingdom and Sweden
Source: Addiction. 2026 Jul 12;121(11):3025–39. doi: 10.1111/add.70526 (PMC13677805; doi:10.1111/add.70526)
Supplement: Supplementary file 1 [file ADD-121-3025-s002.docx]

*Metrebian et al. Overdose reversal through peer intervention: findings from the Naloxone Prospective Outcome Research Study (NalPORS) on the effectiveness of overdose response by people who use opioids in the UK and Sweden.*

**Supplementary material**

Aims and objectives of NaLPORS .

To determine the proportion of participants provided with THN, who witness an opioid overdose and administer naloxone with the intention of reversing an opioid overdose.

To determine the frequency of deaths in the 24 hours (or later if information is available) subsequent to the administration of naloxone.

Other objectives include:

Among community members provided with a supply of THN:

a To determine the daily carriage rate of THN amongst participants supplied with THN

b To determine the proportion of participants supplied with THN who witness an opioid overdose over a 6-month period

To determine the proportion of participants, supplied with THN, who are at risk of experiencing an overdose themselves, who report experiencing an overdose

Of the Witnessed overdose cohort:

To determine the proportion of participants supplied with THN who are carrying THN at a witnessed overdose event

To determine the proportion of participants supplied with THN who witness an opioid overdose, where there is THN present

To determine the proportion of participants supplied with THN, who witness an opioid overdose, where there is THN present and who administer THN to reverse an opioid overdose

To determine the proportion of participants, who witness an opioid overdose, where THN is administered to reverse an opioid overdose, who report a recurrence of respiratory depression within 1-hour of THN administration

To determine the proportion of participants who witness an opioid overdose, where THN is administered to reverse an opioid overdose, who report a second dose of naloxone was administered within 1-hour of administration of the first dose of THN

To determine the proportion of participants who witness an opioid overdose, where THN is administered to reverse an opioid overdose, who report opioid withdrawal symptoms and/or angry (including abusive or hostile) reaction precipitated when THN is administered

To determine the survival rate of individuals having received naloxone at 2 hours post—administration of THN or at arrival of ambulance/medical assistance if this occurs before 2 hours have passed since the THN was administered, as far as this can be ascertained

To determine the proportion of fatal outcomes within a 12-hour period after the witnessed opioid overdose, as far as this can be ascertained.

In case of fatal outcome: to determine what is known about the cause of the fatal outcome (other than fatal overdose), based on any direct or second-hand information available to the participant (e.g., delays in delivery of intervention such that death or irreversible brain damage already occurred, and/or failures by the responder such as failure to call emergency services or failure to administer naloxone, and/or failure of the naloxone to reverse the overdose, and/or failure of the medication device)

To identify the accuracy of diagnosis of a probable opioid overdose by participants supplied with THN and witnessing an overdose

To assess the appropriateness of the response to opioid overdose by participants supplied with THN and witnessing an overdose

Outcomes of NalPORSy

The co-primary outcomes were to determine:

a)	the proportion of participants provided with THN, who witnessed an opioid overdose, and administered naloxone with the intention of reversing an opioid overdose and

b)	the frequency of deaths in the 24 hours (or later when information was available) subsequent to the administration of naloxone.

Secondary outcomes

Among community members provided with a supply of THN:

a) Percentage of participants who report carrying THN (on day of interview)
b) Percentage of participants who witness an opioid overdose over 6-month period
c) Percentage of participants, who are at a risk of experiencing an opioid overdose themselves, who report experiencing an opioid overdose (fatal or non-fatal)”

Of the Witnessed overdose cohort:

d) Percentage of participants who are carrying THN at a witnessed overdose
e) Percentage of participants who report there was THN present
f) Percentage of participants who state there was THN present, and administer THN
g) Percentage of participants who administer THN and report that respiratory depression occurred in the person they resuscitated within one hour of THN administration
h) Percentage of participants who report administering a second dose of THN to a person experiencing an opioid overdose, within 1-hour post administration of first dose of THN
i) Percentage of participants who report that withdrawal symptoms and/ or anger, rage or violence occurred in person they resuscitated with THN.
j) Frequency of each symptom witnessed (at most recent overdose witnessed)
k) Percentage of participants who report that the person receiving naloxone has survived at 2 hours post administration of naloxone, or at arrival of ambulance/medical assistance, if this occurs before 2 hours have passed since the opioid overdose.
l) Percentage of participants who report fatal outcomes within 2 hours of the identification of opioid overdose, or at the arrival of an ambulance/medical assistance, where THN was administered and where THN was not administered.
m) Percentage of participants who correctly diagnose opioid overdose crisis (assessed through multiple response questions). Participants who reported witnessing an overdose were asked to identify, from a list of possible responses, what were the signs that made them or someone else recognize that this was an opioid overdose including: a) key critical signs (loss of consciousness, unresponsiveness /lack of response to calling name or shaking or pain, shallow/absent/slow breathing, blue lips, hands, or feet); b) other signs (deep snoring/ grunting, position of body, needle in arm, small pupils, presence of vomit and collapse); c) Miscellaneous signs/not signs of opioid overdose but might be signs that something is wrong with the individual: (fitting, bloodshot eyes, presence of drooling, agitated behaviour, rapid heartbeat, person reporting not feeling right or other).

Response to the overdose, in addition to naloxone, was collected. This included: tried to wake them verbally (shouted/ called the person’s name), tilted head back, checked for response, checked breathing, put into recovery position, chest compressions, rescue breathing / mouth-to-mouth, cleared airways, checked pulse, ambulance called, ambulance gave naloxone.

**Site Recruitment details**

This study was intended to be conducted in six countries/nations in Europe (European Economic Area (EEA)). Our country eligibility criteria included: (a) substantial opioid use problem, (b) substantial problem of opioid overdose deaths, (c) already providing take-home naloxone, (d) ability to stay in contact and assist with follow-up, (e) ability to report on deaths. Within each country, participating study sites had to fulfil the following eligibility criteria: routinely providing THN, were an active drug treatment facility, needle and syringe programme (NSP) or community/family support group and could reasonably commit to recruiting a substantial number of participants. We aimed to include at least four drug treatment and/or harm reduction services with THN programmes, from each country.

Recruitment sites routinely supplied people with either injectable or nasal naloxone or both. Thus, participants in this study were supplied with whatever formulation type of naloxone was offered at the site. We tried to choose countries and sites which would enable us to achieve a balance of injectable and nasal naloxone.

Due to challenges in recruitment, the study was conducted in 3 countries/nations across the UK (England, Scotland and Wales) and Sweden. Reasons for difficulties in recruitment included challenges in recruiting European countries (e.g., low numbers of reported opioid overdoses, or absence of THN programmes or insecurity of funding for THN programmes).

Countries originally approached but who didn’t participate included:

•	Country A was strongly supportive of the idea of the prospective cohort study but had concerns about whether it was proper to conduct such a study with underpinning funding from the manufacturer of one of the products, and eventually chose not to participate.

•	Country B was keen to participate but had major challenges for the clinical service provision of take-home naloxone and could not be confident about continued provision of this service and hence unsure about whether follow-up could be enabled.

•	Country C and Country D were keen to be involved but we were unable to find a sufficiently centralised system to organise recruitment and track participants, and so we concluded it was not viable within the funds available.

•	Country E was keen to be involved but did not seem to have a system for tracking deaths, or deaths did not occur, and so they did not meet our country eligibility criteria.

•	Country F was appropriate and keen to be involved but then experienced a major change in the illicit drug market and consequently were then experiencing very few overdose events.

•	Country G was keen to be involved but the necessity for supplementary funds for contact and follow-up led us to conclude that it was not realistic to proceed.

Table A. Characteristics of Total cohort (followed-up and not followed-up)(N=1025)

|  | | **Cohort followed-up n=594** | | | **Cohort not followed-up n=431** | | | **Total cohort (N=1025)** | | |
| --- | --- | --- | --- | --- | --- | --- | --- | --- | --- | --- |
|  | | **Total**  **People who have used opioids**  **Groups 1&2**  **n=594** | **Group 1**  **In treatment (Patient/service user in treatment for opioid use disorder)**  **n= 515** | **Group 2**  **Not-in- treatment (Individual uses opioids and not currently in treatment)**  **n= 79** | **Total**  **People who have used opioids**  **Groups 1&2**  **n=431** | **Group 1**  **In treatment (Patient/service user in treatment for opioid use disorder)**  **n= 387** | **Group 2**  **Not-in- treatment (Individual uses opioids and not currently in treatment)**  **n= 44** | **Total**  **People who have used opioids**  **Groups 1&2**  **N=1,025** | **Group 1**  **In- treatment (Patient/service user in treatment for opioid use disorder)**  **N=902** | **Group 2**  **Not-in- treatment (Individual uses opioids and not currently in treatment)**  **N=123** |
| **Countries** | | | | | | | | | | |
| England | | 338 (56.9%) | 335 (65%) | 3 (3.8%) | 270 (62.6%) | 267 (69%) | 3 (6.8%) | 608 (59.3%) | 602 (66.7%) | 6 (4.9%) |
| Sweden | | 171 (28.8%) | 114 (22.1%) | 57 (72.2%) | 21 (4.9%) | 9 (2.3%) | 12 (27.3%) | 192 (18.7%) | 123 (13.6%) | 69 (56.1%) |
| Scotland | | 46 (7.7%) | 38 (7.4%) | 8 (10%) | 76 (17.6%) | 60 (15.5%) | 16 (36.4%) | 122 (11.9%) | 98 (10.9%) | 24 (19.5%) |
| Wales | | 39 (6.6%) | 28 (5.4%) | 11 (14%) | 64 (14.8%) | 51 (13.2%) | 13 (29.5%) | 103 (10%) | 79 (8.8%) | 24 (19.5%) |
| **Type of recruitment site** | | | | | | | | | | |
| Treatment service | | 386 (65%) | 383 (74.4%) | 3 (3.8%) | 297 (69%) | 293 (75.7%) | 4 (9%) | 683 (66.6%) | 676 (74.9%) | 7 (5.7%) |
| Harm reduction | | 208 (35%) | 132 (25.6%) | 76 (96.2%) | 134 (31%) | 94 (24.3%) | 40 (91%) | 342 (33.4%) | 226 (25.1%) | 116 (94.3%) |
| **Participant characteristics** | | | | | | | | | | |
| Age at enrolment | | 45.5; sd. 10 (19.5-71) | 46; sd. 9.8 (21.4-71) | 42.3; sd. 10.9 (19.5-69) | 43; sd 8.8 (20.5-71.2) | 43.3; sd 8.7 (23.3-67) | 40; sd 9.8 (20.5-71.2) | 44.4; sd. 9.6  (19.5-71.2) | 44.8; sd 9.4  (21.3-71) | 41.55; sd 10.5 (19.5-71.2) |
| Gender | |  |  |  |  |  |  |  |  |  |
|  | Male | 443 (75%) | 391 (76.5%) | 52 (65.8%) | 312 (72.7%) | 285 (74%) | 27 (61.4%) | 755 (74%) | 676 (75%) | 79 (64%) |
|  | Female | 148 (25%) | 121 (23.5%) | 27 (34.2%) | 117 (27.3%) | 100 (26%) | 17 (38.6%) | 265 (26%) | 221 (24.5%) | 44 (36%) |
|  | Missing | 3 | 3 | 0 | 2 | 2 | 0 | 5 | 5 | 0 |
| Ethnicity | |  |  |  |  |  |  |  |  |  |
|  | White | 472 (79.6%) | 406 (79%) | 66 (83.5%) | 349 (81.2%) | 311 (80.6%) | 38 (86.4%) | 821 (80%) | 717 (80%) | 104 (85%) |
|  | Black/ African/ Carribean/ Black British | 41 (6.9%) | 39 (7.6%) | 2 (2.5%) | 35 (8.1%) | 33 (8.5%) | 2 (4.5%) | 76 (7%) | 72 (8%) | 4 (3%) |
|  | Mixed/Multiple ethnic group | 36(6.1%) | 29 (5.6%) | 7 (8.9%) | 27 (6.3%) | 23 (6%) | 4 (9.1%) | 63 (6%) | 52 (6%) | 11 (9%) |
|  | Other ethnic groups | 26 (4.4%) | 26 (5.1%) | 0 | 12 (2.8%) | 12 (3.1%) | 0 | 38 (4%) | 38 (4%) | 0 (0%) |
|  | Asian/ Asian British | 14 (2.4%) | 11 (2.1%) | 3 (3.8%) | 6 (1.4%) | 6 (1.6%) | 0 | 20 (2%) | 17 (2%) | 3 (2%) |
|  | Prefer not to say | 4 (<1%) | 3 (<1%) | 1 (1.3%) | 1 (<0.5) | 1 (<0.5%) | 0 | 5 (<1%) | 4 (<1%) | 1 (<1%) |
|  | Missing | 1 | 1 | 0 | 1 | 1 | 0 | 2 | 2 | 0 |
| **Type of naloxone kit supplied at enrolment (potential for multiple kits to be supplied)** | | | | | | | | | | |
|  |  |  |  |  |  |  |  |  |  |  |
|  | Injectable naloxone: pre-filled syringe (Prenoxad) | 346 (56.8%) | 326 (61.4%) | 20 (25.3%) | 302 (67%) | 276 (67.8%) | 26 (59%) | 648 (63.2%) | 602 (66.7%) | 46 (37.4%) |
|  | Nasal naloxone (Nyxoid & Ventizolve) | 254 (41.6%) | 195 (36.7%) | 59 (74.7%) | 142 (31.5%) | 125 (30.7%) | 17 (38.7%) | 396 (38.6%) | 320 (35.5%) | 76 (61.8%) |
|  | Other type of naloxone | 10 (1.6%) | 10 (1.9%) | 0 | 7 (1.5%) | 6 (1.5%) | 1 (2.3%) | 16 (1.6%) | 16 (1.8%) | 0 |

# **NalPORS QUESTIONNAIRE**

**Instructions:** This form is to be completed by research staff for all study participants.

*“Hi [insert name]. My name is [insert name] and I am a researcher from [local site]. As you may remember, in [insert month of recruitment] you agreed to take part in a discussion about overdoses and naloxone use and receive our monthly text messages. I am now calling to ask a few more questions. This questionnaire takes between 15 to 30 minutes to complete. At the end of it you will be reimbursed with a £15 voucher for your time.*

*Is now a good time to talk?*

*The reason for this study is that the number of overdose deaths has been increasing in recent years. We are asking you these questions to understand why this might be happening and what we can do to prevent overdose. Anything you say will be confidential and* *won’t affect your treatment.*

*Your answers are important for us to understand how naloxone is used in the real world.*

*I may ask some questions about situations that may be difficult or upsetting to talk about, if you are uncomfortable at any stage, please let me know and we can stop t or take a break. I will go through a series of fixed questions and I may ask a question you don’t know the answer to, if so, just let me know and we can move on. I will be taking notes while we are talking and at times I may ask you to repeat or clarify. This is so I can be sure I am getting the answers right. First, I will start by asking about naloxone. Is it ok to begin?”*

| ***Interview (researcher only)*** | | | |
| --- | --- | --- | --- |
| a. | Method of interview | 1 | Telephone |
|  |  | 2 | In-person |
|  |  | 3 | Videoconference (e.g. MS Teams, Zoom) |
| b. | Type of follow-up | 1 | Participant initiated |
|  |  | 2 | Standard 6-month follow-up |
|  | | | |
| **Take-home Naloxone Setting and Carriage** | | | |
| 1. | Do you still have your THN kit? [that you received at recruitment] | 0 | No [SKIP TO 10] |
|  |  | 1 | Yes |
|  |  | *999* | *I don’t know* [SKIP TO 10] |
| 2. | Could you tell me where you are right now? (If interview not done in person) | 1 | Home |
|  |  | 2 | Not at home |
|  |  | 777 | Not applicable (interview done in person) |
| 3. | Are you carrying a THN kit with you today? | 0 | No |
|  |  | 1 | Yes |
|  |  | *777* | *Not applicable (no longer has THN kit)* |
|  |  | *888* | *Not done* |
|  |  | *999* | *Unknown* |
| 4. | Where is your THN kit right now? (tick one) | 1 | In my pocket or bag (or attached to a keyring) **that I’m wearing now** |
|  |  | 2 | Private location (home, friend’s home, etc.) |
|  |  | 3 | Public location (street, park, toilet) |
|  |  | 4 | Semi-public location (shelter, hostel, B&B) |
|  |  | 5 | Somewhere else (specify) |
|  |  | *777* | *Not applicable (no longer has THN kit)* |
|  |  | *888* | *Not done* |
|  |  | *999* | *Unknown /* I don’t know where |
| 5. | Is this place near where you or someone else uses drugs? | 0 | No |
|  |  | 1 | Yes |
|  |  | 2 | Sometimes |
|  |  | *777* | *Not applicable* |
|  |  | *888* | *Not done* |
|  |  | *999* | *Unknown* |
| 6. | Which THN kit(s) do you have **right now**? Can you describe what it looks like?  *(Tick all that apply)* | 1 | Single-step naloxone nasal spray: Nyxoid |
|  |  | 2 | Single-step naloxone nasal spray: Ventizolve |
|  |  | 3 | Injectable naloxone: prefilled syringe: Prenoxad |
|  |  | 4 | Injectable naloxone: vial and syringe (e.g. “keyring naloxone”) |
|  |  | 5 | Multi-step naloxone nasal spray (prefilled syringe with screw-on spray) |
|  |  | 6 | Other |
|  |  | *777* | *Not applicable (no longer has THN kit)* |
|  |  | *888* | *Not done* |
|  |  | *999* | *Unknown* |
| 7. | How many minutes would it take you to reach your THN kit if there was an emergency **right** now? | 1 | Less than 2 minutes |
|  |  | 2 | 2-5 minutes |
|  |  | 3 | 6-10 minutes |
|  |  | 4 | More than 10 minutes |
|  |  | *777* | *Not applicable (no longer has THN kit)* |
|  |  | *888* | *Not done* |
|  |  | *999* | *Unknown* |
| 8. | How often in the past week did you have take-home naloxone on you when you left the house? | 1 | *Never* (0 days): |
|  |  | 2 | Sometimes (1-6 days) |
|  |  | 3 | Always (7 days) |
|  |  | *777* | *Not applicable (no longer has THN kit)* |
|  |  | *888* | *Not done* |
|  |  | *999* | *Unknown* |
| 9. | Who is the THN kit for? (tick all that apply) | 1 | Me if I overdose [SKIP TO 11] |
|  |  | 2 | A family member, partner or friend if they overdose [SKIP TO 11] |
|  |  | 3 | A stranger if they overdose (and I happen to be there) [SKIP TO 11] |
|  |  | 4 | For use at the workplace |
|  |  | *777* | *Not applicable (no longer has THN kit) [SKIP TO 10]* |
|  |  | *888* | *Not done* |
|  |  | *999* | *Unknown* |
| 10. | What happened to your last kit? | *1* | Used for an OD |
|  |  | *2* | Lost it |
|  |  | *3* | Stolen |
|  |  | *4* | Gave away |
|  |  | *5* | Expired |
|  |  | *6* | Didn’t want/need it |
|  |  | *7* | Other |
|  |  | *777* | *Not applicable (still has THN kit)* |
|  |  | *888* | Not done |
|  |  | *999* | Unknown |
| *“We will now move on to talk about overdoses and if you have seen any.”* | | | |
|  | | | |
| **Witnessed Opioid Overdose** | | | |
| 11. | Have you seen an opioid overdose in the past x months? [INTERVIEWER STATES MONTH OF ENROLLMENT] | 0 | No [FAMILY/STAFF: SKIP TO 54; PWUO: SKIP TO 46] |
|  |  | 1 | Yes |
|  |  | *777* | *Not applicable* |
|  |  | *888* | *Not done* |
|  |  | *999* | *Unknown* |
| 12. | How many?  *Count all overdoses during the period since enrolment, including any that were previously reported.* |  | *_____ ______ Numerical responses only.* |
| **If this is the participant’s second interview, skip to 46 for PWUO or 54 for family/staff** | | | |
| *Describe the most recent opioid overdose and only one person if multiple people overdosed at the same event* | | | |
| 13. | What is your relationship to the person that overdosed? I am their (Tick only one): | 1 | Partner/spouse |
|  |  | 2 | Parent |
|  |  | 3 | Sibling |
|  |  | 4 | Child |
|  |  | 5 | Other relative |
|  |  | 6 | Friend |
|  |  | 7 | Acquaintance |
|  |  | 8 | Professional (outreach worker, team member from clinic, etc.) |
|  |  | 9 | Other relationship |
|  |  | *777* | *Not applicable / No relationship* |
|  |  | *888* | *Not done* |
|  |  | *999* | *Unknown* |
| 14. A) | Was there a take-home naloxone kit available when the person overdosed? | 0 | No [SKIP TO 16] |
|  |  | 1 | Yes, my own [SKIP TO 14B] |
|  |  | 2 | Yes, someone else’s [SKIP TO 15] |
|  |  | 3 | Yes, mine and someone else’s [SKIP TO 14B] |
|  |  | *777* | *Not applicable* |
|  |  | *888* | *Not done* |
|  |  | *999* | *Unknown /* I don’t know [SKIP TO 16] |
| 14. B) | Which THN kit were you carrying when the person overdosed? | *1* | Single-step naloxone nasal spray: Nyxoid |
|  |  | *2* | Single-step naloxone nasal spray: Ventizolve |
|  |  | *3* | Injectable naloxone: prefilled syringe: Prenoxad |
|  |  | *4* | Injectable naloxone: vial and syringe (e.g. “keyring naloxone”) |
|  |  | *5* | Multi-step naloxone nasal spray (prefilled syringe with screw-on spray) |
|  |  | *6* | Other |
|  |  | *777* | *Not applicable (no longer has THN kit)* |
|  |  | *888* | *Not done* |
|  |  | *999* | *Unknown* |
| 15. | Did you (or someone else) treat the person with take-home naloxone? | 0 | No (THN not given) |
|  |  | 1 | Yes, me |
|  |  | 2 | Yes, someone else |
|  |  | 3 | Yes, me and someone else |
|  |  | *777* | *Not applicable (no THN)* |
|  |  | *888* | *Not done* |
|  |  | *999* | *Unknown* |
| 16. | Did you call the ambulance? | 0 | No [SKIP TO 18] |
|  |  | 1 | Yes, I did |
|  |  | 2 | Somebody else called the ambulance |
|  |  | *777* | *Not applicable* |
|  |  | *888* | *Not done* |
|  |  | *999* | *Unknown /* I don’t know [SKIP TO 18] |
| 17. | Did the ambulance treat the person at the scene? | 0 | No |
|  |  | 1 | Yes |
|  |  | *777* | *Not applicable* |
|  |  | *888* | *Not done* |
|  |  | *999* | *Unknown /* I don’t know |
| 18. | Was the person taken to the hospital? | 0 | No [SKIP TO 21] |
|  |  | 1 | Yes |
|  |  | *777* | *Not applicable* |
|  |  | *888* | *Not done* |
|  |  | *999* | *Unknown /* I don’t know [SKIP TO 21] |
| 19. | Was the hospitalisation due to the opioid overdose? | *0* | *No [SKIP TO 20]* |
|  |  | *1* | *Yes [SKIP TO 21]* |
|  |  | *777* | *Not applicable* |
|  |  | *888* | *Not done* |
|  |  | *999* | *Unknown* |
| 20. | If not for opioid overdose, what was the cause of the hospitalization? Were there other complications?  *(Please write what the participant says and categorise (1-12) after the interview (tick any that apply).*  *Do not read out answers!*  **(IF YES TO ANY, COMPLETE ADR FORM)** | Free text |  |
|  |  | **Interviewer: please categorise based on the response given above:** | |
|  |  |  | |
|  |  | **Concern for individuals’ symptoms/medical condition** | |
|  |  | 1 | **Immune system issue** (Hypersensitivity, Anaphylactic shock; allergic reaction) (à ADR form) |
|  |  | 2 | **Nervous system issue** (Dizziness, Headache, Tremor, shaking) (à ADR form) |
|  |  | 3 | **Cardiac issue** (Fast heartbeat, heart stopped, slow heartbeat, heart attack, heart problems) (à ADR form) |
|  |  | 4 | **Vascular issue** (low or high blood pressure, stroke) (à ADR form) |
|  |  | 5 | **Respiratory, thoracic and mediastinal issue** (Hyperventilation  Pulmonary oedema, coughing up blood, difficulty breathing (feeling like drowning)) (à ADR form) |
|  |  | 6 | **Gastrointestinal issue** (Nausea, Vomiting, Diarrhoea, Dry mouth) (à ADR form) |
|  |  | 7 | **Skin and subcutaneous tissue disorders** (rash) (à ADR form) |
|  |  | 8 | **Other (state)** (à ADR form) |
|  |  | **Other reasons** | |
|  |  | 9 | **Injury from naloxone*** (à ADR form) |
|  |  | 10 | **Incorrect use of naloxone***(à ADR form) |
|  |  | 11 | **Injury as a result of becoming unconscious** (ie. Fall, or hit by car) (à ADR form) |
|  |  | 12 | **Administration to pregnant female*** (à ADR form) |
|  |  | *777* | *Not applicable* |
|  |  | *888* | *Not done* |
|  |  | *999* | *Unknown* |
| 21. | To the best of your knowledge, is the person still alive today? | 0 | No, they died |
|  |  | 1 | Yes, alive [SKIP TO 25] |
|  |  | *777* | *Not applicable* |
|  |  | *888* | *Not done* |
|  |  | *999* | *Unknown /* I don’t know [SKIP TO 25] |
| 22. | Can you recall the exact date of their death? |  | _ _ (dd) _ _ (mm) _ _ _ _ (yyyy) |
| 23. | How soon after the overdose did they die? | 1 | Within 24 hours of the identification of the overdose (or when the ambulance arrived) |
|  |  | 2 | Within 2 hours of the identification of the overdose (or when the ambulance arrived) |
|  |  | 3 | Within 1 hour of the identification of the overdose (or when the ambulance arrived) |
|  |  | 4 | After 24 hours of the identification of the overdose (or when the ambulance arrived) |
|  |  | *777* | *Not applicable* |
|  |  | *888* | *Not done* |
|  |  | *999* | *Unknown /* I don’t know |
| 24. A) | How many minutes after THN was given did the person die? |  | Specify in minutes: |
|  |  | 777 | Not applicable (THN not given) |
|  |  | *888* | *Not done* |
|  |  | *999* | *Unknown /* I don’t know |
| 24 B) | What do you think was the cause of death? | *1* | *Opioid overdose [SKIP TO 25]* |
|  |  | *2* | *Not opioid overdose* |
| 24 C) | If not opioid overdose, what do you think the cause was?  *(Please write what the participant says and categorise (1-12) after the interview (tick any that apply).*  *Do not read out answers!*  **(IF YES TO ANY, COMPLETE ADR FORM)** | Free text |  |
|  |  | **Interviewer: please categorise based on the response given above:** | |
|  |  | **Concern for individuals’ symptoms/medical condition** | |
|  |  | 1 | **Immune system issue** (Hypersensitivity, Anaphylactic shock; allergic reaction) (à ADR form) |
|  |  | 2 | **Nervous system issue** (Dizziness, Headache, Tremor, shaking) (à ADR form) |
|  |  | 3 | **Cardiac issue** (Fast heartbeat, heart stopped, slow heartbeat, heart attack, heart problems) (à ADR form) |
|  |  | 4 | **Vascular issue** (low or high blood pressure, stroke) (à ADR form) |
|  |  | 5 | **Respiratory, thoracic and mediastinal issue** (Hyperventilation  Pulmonary oedema, coughing up blood, difficulty breathing (feeling like drowning)) (à ADR form) |
|  |  | 6 | **Gastrointestinal issue** (Nausea, Vomiting, Diarrhoea, Dry mouth) (à ADR form) |
|  |  | 7 | **Skin and subcutaneous tissue disorders** (rash) (à ADR form) |
|  |  | 8 | **Other (state)** (à ADR form) |
|  |  | **Other reasons** | |
|  |  | 9 | **Injury from naloxone*** (à ADR form) |
|  |  | 10 | **Incorrect use of naloxone***(à ADR form) |
|  |  | 11 | **Injury as a result of becoming unconscious** (ie. Fall, or hit by car) (à ADR form) |
|  |  | 12 | **Administration to pregnant female*** (à ADR form) |
|  |  | *777* | *Not applicable* |
|  |  | *888* | *Not done* |
|  |  | *999* | *Unknown* |
| **[Space for interviewer to acknowledge situation and if participant will continue]** | | | |
|  | | | |
| *“Now we are going to get into some of the details of the overdose.”* | | | |
| **Scene of Overdose** | | | |
| 25. | When did this overdose happen?  *(Code the day as first of the month as default if the exact date is unknown)* |  | _ _ (dd) _ _ (mm) _ _ _ _ (yyyy) |
|  |  | If unable to recall month, within last: | |
|  |  | 1 | Week |
|  |  | 2 | 2 weeks |
|  |  | 3 | 1 month |
|  |  | 4 | 2 months |
|  |  | 5 | 3 months |
|  |  | 6 | 4 months |
|  |  | 7 | 5 months |
|  |  | 8 | 6 months |
|  |  | *777* | *Not applicable (date provided above)* |
|  |  | *888* | *Not done* |
|  |  | *999* | *Unknown /* Don’t remember |
| 26. | What is the gender of the person that overdosed? | 1 | Male |
|  |  | 2 | Female |
|  |  | 3 | Other: |
|  |  | *777* | *Not applicable* |
|  |  | *888* | *Not done* |
|  |  | *999* | *Unknown* |
| 27. | How old was the person that overdosed? | 1 | Under 14 |
|  |  | 2 | 14-18 |
|  |  | 3 | 19 – 24 |
|  |  | 4 | 25 – 44 |
|  |  | 5 | 45 – 64 |
|  |  | 6 | 65 or above |
|  |  | *777* | *Not applicable* |
|  |  | 888 | Not done |
|  |  | 999 | Unknown / Unable to say |
| 28. | Where did the overdose happen? (Tick only one) | 1 | At my home |
|  |  | 2 | At someone else’s home |
|  |  | 3 | In a hostel |
|  |  | 4 | In a public setting (e.g. shopping mall, park, street parking lot, car park, deserted abandoned building, street, in an alleyway) |
|  |  | 5 | A ‘drug using environment’ (where people go to use/buy drugs, such as a ‘crack house’ or a ‘shooting gallery’ |
|  |  | 6 | In a public toilet |
|  |  | 7 | At an addiction service |
|  |  | 8 | Drug consumption room |
|  |  | 9 | Café, restaurant environment (e.g. toilets in a fast food outlet such as McDonald’s) |
|  |  | 10 | Other |
|  |  | *777* | *Not applicable* |
|  |  | *888* | *Not done* |
|  |  | *999* | *Unknown /* I don’t know/I can’t remember |
| 29. | When the person overdosed, do you know which opioids, other drugs, and alcohol they were using? (Tick all that apply)  **RESEARCHER: If no opioids reported, also fill out question 60.** | 1 | Methadone/ buprenorphine from a drug service |
|  |  | 2 | Street methadone/buprenorphine |
|  |  | 3 | Heroin |
|  |  | 4 | Medically prescribed heroin |
|  |  | 5 | Other medically prescribed opioids (e.g. oxycodone) |
|  |  | 6 | Street fentanyl |
|  |  | 7 | Other street opioids (e.g. Oxycodone) |
|  |  | 8 | Other unknown opioid |
|  |  | 9 | Crack cocaine |
|  |  | 10 | Powder cocaine |
|  |  | 11 | Amphetamines |
|  |  | 12 | Street benzodiazepines (e.g. Xanax, Valium) |
|  |  | 13 | Prescribed benzodiazepines |
|  |  | 14 | Herbal cannabis (weed, hashish) |
|  |  | 15 | Synthetic cannabis (spice) |
|  |  | 16 | Alcohol |
|  |  | 17 | Tobacco |
|  |  | 18 | Pills |
|  |  | 19 | Other |
|  |  | *777* | *Not applicable* |
|  |  | *888* | *Not done* |
|  |  | *999* | *Unknown /* I don’t know |
| 30. | Do you know how they used opioids? (Tick all that apply) | 1 | Injected in a vein |
|  |  | 2 | Injected into muscle |
|  |  | 3 | Smoked/chased |
|  |  | 4 | Snorted |
|  |  | 5 | Oral |
|  |  | 6 | Other:______ |
|  |  | *777* | *Not applicable* |
|  |  | *888* | *Not done* |
|  |  | *999* | *Unknown / I don’t know* |
| 31. | How many minutes went by between the opioid use and the overdose? | _____ | Minutes |
|  |  | *777* | *Not applicable* |
|  |  | *888* | *Not done* |
|  |  | *999* | *Unknown / I don’t know* |
| **Opioid Overdose Recognition and Initial Response** | | | |
| 32. | What were the signs that made you (or someone else) recognize the overdose? (Tick all that apply) | 1 | Loss of consciousness |
|  |  | 2 | Unresponsive / Lack of response to calling their name or to shaking or pain |
|  |  | 3 | Shallow/absent/ slow breathing |
|  |  | 4 | Deep snoring/grunting |
|  |  | 5 | Blue lips, hands or feet |
|  |  | 6 | Position of body |
|  |  | 7 | Bloodshot eyes |
|  |  | 8 | Needle in arm |
|  |  | 9 | Presence of drooling |
|  |  | 10 | Small pupils |
|  |  | 11 | Agitated behaviour |
|  |  | 12 | Presence of vomit |
|  |  | 13 | Fitting |
|  |  | 14 | Collapse |
|  |  | 15 | Rapid heartbeat |
|  |  | 16 | Person reported not feeling right |
|  |  | 17 | Other:_________ |
|  |  | *777* | *Not applicable* |
|  |  | *888* | *Not done* |
|  |  | *999* | *Unknown /* I don’t know |
| 33. | Did you (or someone else) do anything to respond to the overdose? If yes, what? (Tick all that apply)  ***(DO NOT read through these possible responses to the individual.** **The list is to capture possible incorrect opioid overdose responses**). | 1 | Tilted head back (QSG) |
|  |  | 2 | Checked for response |
|  |  | 3 | Checked breathing |
|  |  | 4 | Put into recovery position (QSG) |
|  |  | 5 | Chest compressions |
|  |  | 6 | Rescue breathing / mouth to mouth |
|  |  | 7 | Cleared airways |
|  |  | 8 | Checked pulse |
|  |  | 9 | Ambulance called (QSG) |
|  |  | 10 | Ambulance gave naloxone |
|  |  | 11 | Tried to wake them verbally (shouted/ called the person’s name) |
|  |  | 12 | Tried to wake them physically (by shaking, slapping?) |
|  |  | 13 | I asked if they wanted me to use naloxone |
|  |  | 14 | **Injecting a stimulant drug*** |
|  |  | 15 | **Injecting ice water/milk*** |
|  |  | 16 | **Placing in the bath/shower*** |
|  |  | 17 | **Used ice to revive (without injecting). E.g. placed on neck, testicles, thigh, stomach, chest, down back of shirt)*** |
|  |  | 18 | **Walking person around*** |
|  |  | 19 | Nothing |
|  |  | 20 | Other |
|  |  | *777* | *Not applicable* |
|  |  | *888* | *Not done* |
|  |  | *999* | *Unknown /* I don’t know |

| Based on question 15 (THN given or not given) go to the following question: | |
| --- | --- |
| IF THN not given: SKIP TO 34 | IF THN given: SKIP TO 36 |

| *“You had mentioned that take-home naloxone was not given..”* | | | |
| --- | --- | --- | --- |
| **Take-Home Naloxone NOT Given** | | | |
| 34. | What was the main reason take-home naloxone was NOT given? (Tick only one) | 1 | Didn’t have it on me (not carrying THN) |
|  |  | 2 | No longer possessed a THN kit (lost or previously used) |
|  |  | 3 | Didn’t know how to use it |
|  |  | 4 | Afraid to use it |
|  |  | 5 | Panicked |
|  |  | 6 | Thought the person would be angry or aggressive |
|  |  | 7 | Someone else intervened |
|  |  | 8 | Kit malfunction/pieces missing |
|  |  | 9 | Person recovered |
|  |  | 10 | Person had died |
|  |  | 11 | Couldn’t assemble kit |
|  |  | 12 | Injectable naloxone: Did not have access to a clean needle (e.g. keyring naloxone) |
|  |  | 13 | Kit expired |
|  |  | 14 | I judged it was not necessary at that point in time |
|  |  | 15 | Other |
|  |  | *777* | *Not applicable* |
|  |  | *888* | *Not done* |
|  |  | *999* | *Unknown* |
| 35. | How many minutes did you stay with the person who had overdosed? |  | Minutes |
|  |  | *777* | *Not applicable* |
|  |  | *888* | *Not done* |
|  |  | *999* | *Unknown /* I don’t know |
|  | | | |

| *“You mentioned that take-home naloxone was given during the overdose. I’d like to ask you some details about that now. Its ok if you don’t have the answers for every question. We can move on.”* | | | |
| --- | --- | --- | --- |
| **Take-Home Naloxone Given** | | | |
| 36. | What kit was used?  *(tick all that apply)* | 1 | Single-step naloxone nasal spray: Nyxoid |
|  |  | 2 | Single-step naloxone nasal spray: Ventizolve |
|  |  | 3 | Single-step naloxone nasal spray: Other |
|  |  | 4 | Injectable naloxone: prefilled syringe: Prenoxad |
|  |  | 5 | Injectable naloxone: vial and syringe (e.g. “keyring naloxone”) |
|  |  | 6 | Multi-step naloxone nasal spray (prefilled syringe with screw-on spray) |
|  |  | *777* | *Not applicable* |
|  |  | *888* | *Not done* |
|  |  | *999* | *Unknown* |
| 37. | Was more than 1 ~~dose~~ administration of naloxone given within 1 hour of identifying the overdose? (as defined by product package instructions) | 0 | No |
|  |  | 1 | Yes |
|  |  | *777* | *Not applicable* |
|  |  | *888* | *Not done* |
|  |  | *999* | *Unknown / I don’t know* |
| 38. | **BASED ON Q37:**  How much naloxone did you give? | **38.1 FIRST ADMINISTRATION** | |
|  |  | 1 | PRE-FILLED SYRINGE: 1/5^th^ of syringe (0.4mg) |
|  |  | 2 | PRE-FILLED SYRINGE: 2/5ths of syringe |
|  |  | 3 | PRE-FILLED SYRINGE: 3/5ths of syringe |
|  |  | 4 | PRE-FILLED SYRINGE: 4/5ths of syringe |
|  |  | 5 | PRE-FILLED SYRINGE: Whole syringe |
|  |  | 6 | VIAL: Whole vial (0.4mg) |
|  |  | *7* | VIAL: Less than whole vial |
|  |  | *8* | VIAL: More than one vial |
|  |  | 9 | NASAL SPRAY: 1 spray |
|  |  | 10 | NASAL SPRAY: 2 sprays |
|  |  | 11 | NASAL SPRAY: More than 2 sprays |
|  |  | *777* | *Not applicable* |
|  |  | *888* | *Not done* |
|  |  | *999* | *Unknown /* Don’t know |
|  |  | **38.2 SECOND ADMINISTRATION (**same drop-down menu) IF ‘NO’ TO Q37 THEN SKIP TO Q41 | |
|  |  | **38.3 THIRD ADMINISTRATION** (same drop-down menu) IF ‘NO’ TO Q37 THEN SKIP TO Q41 | |
|  |  | **38.4 FOURTH ADMINISTRATION** (same drop-down menu) IF ‘NO’ TO Q37 THEN SKIP TO Q41 | |
|  |  | **38.5 FIFTH ADMINISTRATION** (same drop-down menu) IF ‘NO’ TO Q37 THEN SKIP TO Q41 | |
| 39. | **Repeat ADMINISTRATION ONLY:**  How many minutes did you wait in between doses before giving more naloxone? |  | Minutes |
|  |  | *777* | *Not applicable* |
|  |  | *888* | *Not done* |
|  |  | *999* | *Unknown /* I don’t know |
| 40. | **Repeat ADMINISTRATION ONLY:**  Why did you decide to give the person another dose of naloxone? (Tick all that apply) | 1 | There was no improvement in 2-3 minutes (e.g. not responding or breathing) |
|  |  | 2 | Overdose symptoms came back |
|  |  | 3 | It was the recommended time I was taught in the training |
|  |  | 4 | The ambulance had not arrived yet |
|  |  | 5 | To be on the safe side |
|  |  | 6 | I was panicking |
|  |  | 7 | I didn’t think it would do any harm |
|  |  | 8 | They had died |
|  |  | 9 | Other |
|  |  | *777* | *Not applicable* |
|  |  | *888* | *Not done* |
|  |  | *999* | *Unknown /* I don’t know/can’t remember |
| 41. | Did you notice any reaction to the THN? If yes, what were the reactions? (Tick all that apply)  **(DO NOT read out these possible reactions to the individual.**)  ***IF POSITIVE RESPONSE TO 15-19 COMPLETE ADR FORM** | 0 | No symptoms |
|  |  | 1 | Fast heartbeat |
|  |  | 2 | Muscle cramps / muscle pain |
|  |  | 3 | Runny nose |
|  |  | 4 | Watery eyes (tears) |
|  |  | 5 | Nausea |
|  |  | 6 | Vomiting |
|  |  | 7 | Diarrhoea |
|  |  | 8 | Yawning |
|  |  | 9 | Sweating |
|  |  | 10 | Confusion (short term memory loss, dazed, uncertain of events) |
|  |  | 11 | Agitated (irritable, complaining, moody, unable to stay still, left the scene as quickly as able/possible, wanted to get more drugs as soon as possible) |
|  |  | 12 | Angry (at me, others, shouting and visibly upset/displeased) |
|  |  | 13 | Rage (verbal abuse, threats of violence, uncontrollable temper) |
|  |  | 14 | Complained a lot (to me or others about loss of drugs/ wanted to get more drugs as soon as possible) |
|  |  | 15 | Allergic reaction (anaphylactic shock)* |
|  |  | 16 | Shaking (tremor)* |
|  |  | 17 | Heart problems (slow heart rate, heart stopped, cardiac arrest)* |
|  |  | 18 | Hyperventilated * |
|  |  | 19 | Signs of pulmonary oedema (coughing up blood, difficulty breathing, feeling like ‘drowning’)* |
|  |  | 20 | Other (specify): |
|  |  | *777* | *Not applicable* |
|  |  | *888* | *Not done* |
|  |  | *999* | *Unknown /* I don’t know |
| 42. | If yes to any reactions, state which reaction, describe it, and how long in minutes it was experienced. | 1 | Reaction:  Describe:  Minutes: |
|  |  | 2 | Reaction:  Describe:  Minutes |
|  |  | 3 | Reaction:  Describe:  Minutes |
|  |  | 4 | Reaction:  Describe  Minutes |
|  |  | 5 | Reaction:  Describe:  Minutes |
|  |  | *777* | *Not applicable* |
|  |  | *888* | *Not done* |
|  |  | *999* | *Unknown* |
| 43. | Within 1 hour of giving naloxone, did the person’s breathing become shallow or absent again? | 0 | No |
|  |  | 1 | Yes |
|  |  | *777* | *Not applicable /* I can’t answer that as I didn’t see them an hour later |
|  |  | *888* | *Not done* |
|  |  | *999* | *Unknown /* I don’t know (because I didn’t ask but was with them) |
| 44. | Within 1 hour of their overdose, did the person go on to use heroin (or non-prescribed opioids) again? | *0* | *No* |
|  |  | *1* | *Yes* |
|  |  | *777* | *Not applicable /* I can’t answer that as I didn’t see them an hour later |
|  |  | *888* | *Not done* |
|  |  | *999* | *Unknown /* I don’t know (because I didn’t ask/see – although I was with them |
| 45. | Where there any problems using THN kit? If YES, tick all that apply.  **(DO NOT read out these possible reactions to the individual.**) | 0 | No |
|  |  | 1 | It broke |
|  |  | 2 | The naloxone discharged/came out before I was ready too soon/ sprayed into the air |
|  |  | 3 | The needle was missing from the kit |
|  |  | 4 | Did not have access to a clean needle (e.g. “keyring naloxone”) |
|  |  | 5 | Another part of the kit was missing |
|  |  | 6 | The kit was damaged |
|  |  | 7 | I didn’t know how to use it |
|  |  | 8 | I couldn’t put it together |
|  |  | 9 | Couldn’t open it |
|  |  | 10 | Other |
|  |  | *777* | *Not applicable* |
|  |  | *888* | *Not done* |
|  |  | *999* | *Unknown* |

| THE FOLLOWING QUESTIONS ARE FOR PWUO ONLY! (FAMILY & STAFF: SKIP TO 54)  *“I would now like to ask you some questions about you and any personal experience with opioids or overdoses."* | | | |
| --- | --- | --- | --- |
| **Personal Opioid Use and Overdose** | | | |
| 46. | Have you used opioids in the past 4 weeks? | 0 | No |
|  |  | 1 | Yes, prescribed to me (e.g. methadone prescription) |
|  |  | 2 | Yes, not prescribed to me (e.g. heroin) |
|  |  | 3 | Yes, both |
|  |  | *777* | *Not applicable* |
|  |  | *888* | *Not done* |
|  |  | *999* | *Unknown* |
| 47. | Have you ever overdosed on opioids? | 0 | No [SKIP TO 54] |
|  |  | 1 | Yes |
|  |  | *777* | *Not applicable* |
|  |  | *888* | *Not done* |
|  |  | *999* | *Unknown* |
| 48. | How many times have you overdosed on opioids in the past x months? [INTERVIEWER STATES MONTH OF ENROLLMENT] | ____ ____ (Numerical responses only)  [IF MULTIPLE, DISCUSS THE MOST RECENT] | |
| 49. | Did someone treat you with take-home naloxone during your last opioid overdose?  (To be completed only if the OD happened in the past x months) | 0 | No |
|  |  | 1 | Yes |
|  |  | 2 | I don’t know |
|  |  | *777* | *Not applicable* |
|  |  | *888* | *Not done* |
|  |  | *999* | *Unknown* |
| 50. | How did you respond to getting take-home naloxone? (Tick all that apply)  **(DO NOT read out these possible reactions to the individual.**)  ***IF POSITIVE RESPONSE TO 15-19 COMPLETE ADR FORM** | 0 | No symptoms |
|  |  | 1 | Fast heartbeat |
|  |  | 2 | Muscle cramps / muscle pain |
|  |  | 3 | Runny nose |
|  |  | 4 | Watery eyes (tears) |
|  |  | 5 | Nausea |
|  |  | 6 | Vomiting |
|  |  | 7 | Diarrhoea |
|  |  | 8 | Yawning |
|  |  | 9 | Sweating |
|  |  | 10 | Confusion (short term memory loss, dazed, uncertain of events) |
|  |  | 11 | Agitated (irritable, complaining, moody, unable to stay still, left the scene as quickly as able/possible, wanted to get more drugs as soon as possible) |
|  |  | 12 | Angry (at me, others, shouting and visibly upset/displeased) |
|  |  | 13 | Rage (verbal abuse, threats of violence, uncontrollable temper) |
|  |  | 14 | Complained a lot (to me or others about loss of drugs/ wanted to get more drugs as soon as possible) |
|  |  | 15 | Allergic reaction (anaphylactic shock)* |
|  |  | 16 | Shaking (tremor)* |
|  |  | 17 | Heart problems (slow heart rate, heart stopped, cardiac arrest)* |
|  |  | 18 | Hyperventilated * |
|  |  | 19 | Signs of pulmonary oedema (coughing up blood, difficulty breathing, feeling like ‘drowning’)* |
|  |  | 20 | Other (specify) |
|  |  | *777* | *Not applicable* |
|  |  | *888* | *Not done* |
|  |  | *999* | *Unknown /* I don’t know |
| 51 | Were you treated by ambulance paramedics? | *0* | *No* |
|  |  | *1* | *Yes* |
|  |  | *777* | *Not applicable* |
|  |  | *888* | *Not done* |
|  |  | *999* | *Unknown* |
| 52.a | Were you taken to the hospital? | 0 | No [SKIP TO 54] |
|  |  | 1 | Yes |
|  |  | *777* | *Not applicable* |
|  |  | *888* | *Not done* |
|  |  | *999* | *Unknown /* I don’t know [SKIP TO 54] |
| 52. b | Was your hospitalization due to the opioid overdose? | 0 | No |
|  |  | 1 | Yes |
| 53. | If not for opioid overdose, what was the cause of the hospitalization? Were there other complications?  ***IF POSITIVE RESONSE, COMPLETE ADR FORM** | Free text |  |
|  |  | **Interviewer: please categorise based on the response given above:** | |
|  |  |  | |
|  |  | 1 | **Immune system issue** (Hypersensitivity, Anaphylactic shock; allergic reaction) * |
|  |  | 2 | **Nervous system issue** (Dizziness, Headache, Tremor, shaking) |
|  |  | 3 | **Cardiac issue** (Fast heartbeat, heart stopped, slow heartbeat, heart attack, heart problems) * |
|  |  | 4 | **Vascular issue** (low or high blood pressure, stroke) * |
|  |  | 5 | **Respiratory, thoracic and mediastinal issue** (Hyperventilation  Pulmonary oedema, coughing up blood, difficulty breathing (feeling like drowning)) * |
|  |  | 6 | **Gastrointestinal issue** (Nausea, Vomiting, Diarrhoea, Dry mouth) * |
|  |  | 7 | **Skin and subcutaneous tissue disorders** (rash) * |
|  |  | 8 | **Other (state) *** |
|  |  | **Other reason** | |
|  |  | 9 | **Injury from naloxone*** |
|  |  | 10 | **Incorrect use of naloxone*** |
|  |  | 11 | **Injury as a result of becoming unconscious** (ie. Fall, or hit by car) |
|  |  | 12 | **Administration to pregnant female*** |

| *“The last section I would like to discuss now with you is about take-home naloxone training.”* | | | | |
| --- | --- | --- | --- | --- |
| **Take-Home Naloxone Training** | | | | |
| 54. | What was covered during your THN training? (Tick all that apply) | | 1 | Call an ambulance |
|  |  |  | 2 | Recognising an opioid overdose |
|  |  |  | 3 | Check breathing |
|  |  |  | 4 | Put into the recovery position |
|  |  |  | 5 | Giving naloxone |
|  |  |  | 6 | Giving a first dose of naloxone & waiting for a response |
|  |  |  | 7 | Give repeat doses if no response |
|  |  |  | 8 | Knowing when to give a second dose (e.g. the number of minutes before and after first dose) |
|  |  |  | 9 | Giving chest compressions & rescue breaths |
|  |  |  | 10 | Staying with the person until ambulance arrives |
|  |  |  | 11 | Managing signs of withdrawal |
|  |  |  | 12 | Managing signs of anger |
|  |  |  | 13 | Learning how to communicate without antagonising person recovering from overdose |
|  |  |  | 14 | Tilt the head back |
|  |  |  | 15 | Not trained |
|  |  |  | 16 | Overdose prevention training (safe injection practices, avoiding mixing drugs, etc.) |
|  |  |  | 777 | Not applicable |
|  |  |  | *888* | *Not done* |
|  |  |  | *999* | *Unknown / Don’t remember* |
| 55. | From your THN training, what did you find most useful? | | 1 | Training / in-person conversation (e.g. dosing, assembly, OD recognition) |
|  |  |  | 2 | Training (video) |
|  |  |  | 3 | Leaflet/ written materials |
|  |  |  | 4 | App |
|  |  |  | 5 | Pictures on naloxone packaging (ex. Prenoxad illustrations, Nyxoid quick start guide) |
|  |  |  | 6 | Nothing (didn’t find it helpful) |
|  |  |  | *777* | *Not applicable* |
|  |  |  | *888* | *Not done* |
|  |  |  | *999* | *Unknown / Not sure* |
| ***For those who received Nyxoid only:*** | | | | |
| 56. | | *Have you read the instructions in the pictures on the Nyxoid pack?* | 0 | No |
|  |  |  | 1 | Yes |
|  |  |  | *777* | *Not applicable (no Nyxoid)* |
|  |  |  | *888* | *Not done* |
|  |  |  | *999* | *Unknown /* I don’t remember |
| 57. | | *Have you read the patient information card?* | 0 | No |
|  |  |  | 1 | Yes |
|  |  |  | *777* | *Not applicable (no Nyxoid)* |
|  |  |  | *888* | *Not done* |
|  |  |  | *999* | *Unknown /* I don’t remember |
| 58. | | *Have you read the package leaflet inside the Nyxoid pack?* | 0 | No |
|  |  |  | 1 | Yes |
|  |  |  | *777* | *Not applicable (no Nyxoid)* |
|  |  |  | *888* | *Not done* |
|  |  |  | *999* | *Unknown /* I don’t remember |
| 59. | | *Have you watched the video showing how to give Nyxoid?* | 0 | No |
|  |  |  | 1 | Yes |
|  |  |  | *777* | *Not applicable (no Nyxoid)* |
|  |  |  | *888* | *Not done* |
|  |  |  | *999* | *Unknown* |
| **RESEARCHER: If no opioids reported, also fill out question 60.** | | | | |
| 60. | | *Has the participant given reason to believe the overdose discussed in this interview was not from opioids? If yes, describe rationale.* | 0 | No |
|  |  |  | 1 | Yes (specify): |
|  |  |  | *777* | *Not applicable* |
|  |  |  | *888* | *Not done* |
|  |  |  | *999* | *Unknown* |

| *“We have now reached the end of the questionnaire…. Before we finish our call, we do have one final question for you:* | | | |
| --- | --- | --- | --- |
| 61. | *Because you’ve witnessed an overdose, a member of our research team may want to contact you about taking part in a detailed telephone interview. During this interview, you may be asked similar questions to the ones you’ve just answered, however the focus of this second interview is to collect more details about the witnessed overdose event. This will still be part of our study and still confidential. Are you happy for your contact details to be passed on to them?* | *0* | *No* |
|  |  | *1* | *Yes* |
|  |  | *777* | *Not applicable (no opioid overdose witnessed)* |
| *Thank you for participating in our study, we appreciate you taking the time to talk to us.*  *Do you need to get a new THN kit? [*Interviewer instructs on where to obtain new kit]  [Interviewer to review reimbursement plan]  *We know overdose can be a traumatic thing to witness, if you would like, we can get you in contact with someone in your area who can talk to you about your experiences. “* | | | |
